# Blockchain-enhanced federated learning for IoT security and privacy using the GSR-C2N model

**DOI:** 10.1038/s41598-026-52361-6

**Published:** 2026-06-10

**Authors:** Shahnawaz Ahmad, Mohd. Aquib Ansari, Arvind Mewada, Gulrej Ahmed

**Affiliations:** 1 School of Computer Science Engineering and Technology, Bennett University, Greater Noida, 201310 India; 2 School of Computing Science & Engineering, Galgotias University, Greater Noida, 203201 India; 3 Manipal University Jaipur, Jaipur, 303007 Rajasthan India

**Keywords:** Federated Learning, Blockchain, IoT Security, Privacy Preservation, GSR-C2N, Smart Cities, Healthcare, Computational biology and bioinformatics, Engineering, Mathematics and computing

## Abstract

The rapid proliferation of Internet of Things devices has intensified the demand for security, privacy-preserving, and scalable machine learning solutions. Federated Learning (FL) supports the decentralized training of models across distributed devices without transporting raw data, whereas blockchain provides a trusted, transparent mechanism for integrity and reaching consensus. The paper explains an FL framework that incorporates blockchain technology, based on the GSR-C2N model for identifying crypto-mining malware. It is demonstrated that the system’s feature extraction and optimization processes are optimized to address security problems in IoT by utilizing blockchain to verify model updates and build trust and privacy. The given structure has demonstrated superiority to the current practices. This model was 96.85% accurate and 97.51% specific on the crypto-mining malware data set using 10-fold cross-validation, making it applicable to smart healthcare and smart city applications with IoT-based systems. In addition, when combined with regulatory compliance, homomorphic encryption can strengthen data and privacy management, underscoring the model’s effectiveness in advanced IoT systems.

## Introduction

The IoT has advanced technological ecosystems, with smart cities and automated industries among the many examples of its impact. The billions of interconnected devices demand complex machine learning privacy solutions due to the increased sensitivity of their data. The classical approach of consolidating all data in a centralized server to train a model poses serious risks, such as data leakage, regulatory violations, and constraints on scaling and granularity. FL is a promising paradigm to maintain the security of sensitive information in the eyes of intruders to minimize chances of external damage and aid in safe data management in the process of training IoT device models^1^. FL offers a wide range of device options. The establishment of trust, coordination of devices, and security of the training process against malicious attackers are imperative problems that must be addressed within the dynamic, decentralised context of large-scale FL IoT networks^2^.

The current study is inspired by the fact that the growing security and privacy needs in IoT systems are not sufficiently addressed by available solutions. The increased number of IoT devices has brought more complex threats, such as cryptojacking^3^ and data manipulation, that exploit resource-constrained nodes and centralised vulnerabilities^4^. The graph-based feature extraction and SparRaven optimisation are shown to be efficient within the GSR-C2N framework, which is designed to detect crypto-mining malware^5^. This drives it to adopt IoT, where data is frequently structured, relational, and distributed. Moreover, the inclusion of blockchain as a layer of trust allows recording updates to an FL model in a transparent, irreversible way, eliminating the accountability loopholes of the traditional FL systems. Such an integration of FL, blockchain, and GSR-C2N contributes to IoT security and to development in critical areas such as smart healthcare and Industry 5.0.

The functionality of graph-based feature extraction and SparRaven optimisation was shown to be efficient within the GSR-C2N framework, which was designed to detect crypto-mining malware^5^. It is now well-suited for IoT security applications, with specific adaptations to meet real-time streaming requirements, device heterogeneity, and resource constraints (as elaborated in Sect. 3.3).

Nevertheless, FL and blockchain implementations in the IoT setting have several operational limitations. FL experiences model drift and synchronisation inefficiencies due to asynchronous training across heterogeneous devices. The blockchain algorithms, especially proof-of-work, impose excessive computational requirements that are not well-suited to the IoT environment^6^. Privacy is also a concern, as updates to FL models can be vulnerable to gradient inversion attacks, and blockchain transparency can compromise confidentiality. Additionally, many distributed learning systems lack an inherent mechanism to address ethical, regulatory, and interpretability needs, including those under GDPR and HIPAA^7^.

To address these shortcomings, this paper recommends a blockchain-integrated FL architecture for IoT security that leverages the capabilities of both technologies. Based on the GSR-C2N model, SparRaven optimisation is applied to the graph-based CNN architecture^8^ to improve feature initialization and learning performance. Blockchain is used for the safe aggregation and verification of model updates via proof-of-stake (PoS) consensus^9^, whereas homomorphic encryption is used to preserve data privacy. The suggested structure is scalable, understandable, and resilient to contemporary IoT threats, bridging the gap between hypothetical innovation and real implementation. The novelty of this work lies not merely in the combination of existing technologies but rather in their adaptation within the context of the IoT, specifically, to suit a heterogeneous environment with limited resources. We adapt the GSR-C2N model’s graph-based CNN with targeted modifications for real-time streaming and device heterogeneity, enabling relational feature extraction that captures inter-device dependencies missed by conventional CNNs or LSTMs. We introduce SparRaven, a hybrid metaheuristic (SSA for global exploration + RRO for local refinement), which provides faster convergence and better generalisation under non-IID data compared to standard optimisers or single metaheuristics. We integrate a lightweight PoS blockchain with selective Paillier homomorphic encryption in a fully decentralised FL loop, achieving tamper-proof verification and strong privacy with quantified low overhead (detailed in Sect. 4), unlike many prior FL-blockchain works that either incur high energy costs or lack graph-relational modelling.

This combination addresses several critical shortcomings in existing solutions, including the inability to properly handle the diversity of IoT data, excessive computational and privacy-related burdens, and inadequate robustness against IoT-FL attacks. A formal complexity analysis is provided in Sect.  3.5. The main contributions of this work are as follows:

- We created a new system architecture using GSR-C2N to extract features of graph-based threat detection and use SparRaven to optimize this extraction in the FL paradigm in order to identify threats in IoT networks with reduced costs.
- We incorporate blockchain as a standardized trust system, proof-of-stake consensus to assure data integrity and induce cooperation of participants with less energy consumption.
- We protect privacy by homomorphic encryption, which allows aggregating encrypted model updates (in a secure manner) without violating regulatory and ethical compliance requirements in the IoT applications, including smart healthcare, smart cities, and Industry 5.0.

The framework is tested on an adapted crypto-mining malware dataset and achieves 96.85% accuracy and 97.51% specificity in 10-fold cross-validation, demonstrating its usefulness in protecting complex IoT systems.

The rest of the paper is structured as follows: In Section I, IoT security issues are raised, and the blockchain-enhanced FL framework is introduced, with reference to a GSR-C2N model. Section II provides a review of the related literature on FL, blockchain, and IoT security. Section III provides the description of the proposed model, its main parts, Block Transaction Controller, Graph CNN, SparRaven Optimizer, Blockchain Layer and Homomorphic Safeguarding, and the corresponding algorithm. Section IV includes information on implementation and performance analysis. Section V is about the applications, scalability, privacy and regulation implications of the framework. Section VI wraps up the paper with some insights and outlines future work, including asynchronous FL, fairness considerations, novel cryptographic methods, and energy-saving consensus.

## Related work

FL has become a sophisticated privacy-preserving paradigm that enables collaborative model training without storing raw data centrally, thereby overcoming the security and performance challenges in distributed settings^1^. Its applicability spans various fields, including cryptomining detection, intrusion prevention, malware analysis, and blockchain-based security solutions. Current strategies in the fields show significant improvements, though they also reveal some severe shortcomings that FL can address (Table 1). For example, Gangwal et al.^10^ proposed an HPC-based covert cryptomining detection framework that was highly efficient at certain mining operations but could not scale across different data sets, a problem FL aims to address by using distributed nodes for effective model training^6^. Otherwise, Nagararaju et al.^11^ used Grey Wolf Optimization with deep neural networks (DNNs) to achieve better intrusion detection, but still, there is a risk of falling into local optima. FL has the capacity to address such concerns through the aggregation of diverse and decentralized models to enhance generalization without interfering with privacy^2^. The alternative methods, such as the Crypto Aegis approach of Pastor et al.^12^, are better at detecting attack-specific strategies, such as sponge and fast pool mining, but are less applicable to a broader threat scenario, which limits what one can do with FL integration.

**Table 1:** Comparative analysis of techniques with federated learning potential.

Ref.	Focus area	Methodology	Strengths	Limitations	FL potential
^10^	Covert Cryptomining Detection	High-performance computing (HPC)	Efficient for specific mining detection	Effective only for small datasets	High: FL could train on distributed datasets without centralising sensitive mining data.
^11^	Intrusion Detection Systems (IDS)	Grey Wolf Optimisation + Deep Neural Network	Handles complex features with high accuracy	Falls into local optima	Moderate: FL could reduce overfitting by training on diverse local data.
^12^	Crypto jacking Detection	ML-based Crypto Aegis Method	Detects sponge attacks and faster pool mining	Limited to specific attack types	Low: FL’s benefits are limited for highly specialised detection models.
^13^	Kubernetes Pod Anomaly Detection	ML with Pod Re-moval Strategy	Identifies pod-level anomalies	Shared hosting introduces security risks	High: FL could support learning across multiple clusters without exposing sensitive pod data.
^14^	Malware Detection in Crypto	RNN with 5 LSTMs + 10-fold CV	Effective temporal modelling, deep memory capability	Limited to LSTM-based architectures	Moderate: FL could support generalisation by training on decentralised malware data.
^15^	Crypto-mining Activity Monitoring	DL/ML with Tstat feature extraction(51 features)	Differentiates crypto traffic	The feature set lacks robustness alone	Moderate: FL could enhance representation by aggregating diverse local features.
^16^	Ransomware Payment Classification	SNN + ODT on Bitcoin Network	Supports online processing and signal detection	Highly sensitive to input noise	Low: Model sensitivity limits the gains from FL.
^4^	Blockchain Authentication	Homomorphic Encryption + Trust Model	High scalability, strong privacy	Lacks consensus robustness	Low: FL is not directly applicable to blockchain consensus layers.
^17^	Decentralised Transactions	Blockchain-Based Energy Exchange	Improves integrity, reduces disparity	High privacy risk from transaction transparency	Low: FL helps with privacy, but does not eliminate fundamental blockchain transparency issues.
^18^	Parallel Mining Optimisation	PSO + Proof-of-Work	Optimises resource use, improves throughput	Energy-intensive, complex implementation	Low: FL is not suitable for optimisation tasks within consensus mining.
^19^	Cyber Threat Detection	Parallel Stacked LSTM + Multi- head Attention	Effective detection and decision-making	Weak performance in multi-threat environments	Moderate: FL can improve generalisation across diverse threat patterns.

Outstanding studies point to comparable trade-offs in cybersecurity applications. Kubernetes anomaly detection based on pod removal strategies studied by Karn et al.^13^ enhances anomaly detection, but also introduces more risks in shared hosting environments, which is alleviated by FL through secure, decentralized collaboration between clusters without exposing sensitive data^20^. Yazdinejad et al.^14^ presented an LSTM-based RNN architecture with powerful temporal modeling and a high level of flexibility in the area of cryptocurrency malware detection, but it relies on sequential architectures, which reduces its flexibility. Heterogeneous information sources spread across various settings can be learned with FL integration, thereby improving its adaptability^21^. Caprolu et al.^15^ examined the topic of cryptomining traffic monitoring using statistical analysis and deep learning, but the robustness of the features remains an issue that can be addressed by federating the aggregation of multiple feature sets. On the other hand, some uses of AI in practice, e.g., ransomware payment classification with spiking neural networks (SNN) and orthogonal decision trees^16^, are highly sensitive to noise, which makes FL less effective when the difference between input data is high.

Recent developments in the field of IoMT security are also a compelling force behind the absence of our proposed integration of federated learning and blockchain. Shambharkar et al.^22^ proposed an XAI-based multi-attention Deep CRNN framework to detect cyberattacks in IoMT scenarios and reported significant results in precision and explainability using attention mechanisms. In the same manner, Sharma^23^ investigated enhancing the security of IoMT using the latest intrusion-detection frameworks and advanced deep-learning technologies, demonstrating high detection rates and overcoming the specific limitations of medical equipment. In an extension of the layered-based defence, Sharma and Shambharkar^24^ came up with a multi-layered security architecture to IoMT systems that incorporates an all-time key management approach, decentralized storage, and a reliable intrusion detection model, focusing on resistance against complex attacks. Furthermore, Sharma^25^ proposed the SA-GBO-ODBN model, which combines blockchain and deep learning to process secure data and support healthcare diagnosis, emphasising the compatibility of blockchain immutability with optimal neural structures. These are furthered by our GSR-C2N blockchain-based FL framework, which facilitates model training across a distributed IoT network without centralising sensitive data and leverages graph-based relational feature extraction and SparRaven optimisation to perform better in heterogeneous IoT environments.

Security frameworks involving blockchain also have the opportunities and limitations of FL integration. Homomorphic encryption-based authentication schemes and trust-based authentication schemes^4^ are more privacy- and scalability-friendly but face limitations in achieving consensus. The FL does not add much value in such domains, because it focuses on data privacy rather than the strength of the consensus^26^. Likewise, decentralized energy exchange schemes, which have been introduced using blockchain^17^, enhance the integrity of transactions, but compromise privacy since transparency is guaranteed through transparent logs that FL can partially address by using privacy-preserving collaborative learning without central data storage^27^. Proof-of-work mining methods such as Particle Swarm Optimization^18^, though useful in throughput optimization, are algorithmic in nature, not data-driven, and FL cannot be used. Cyber threat detection systems based on stacked LSTMs with multi-head attention^19^, on the other hand, perform well in single-threat scenarios but fail in multi-threat scenarios, which can be improved in FL because it combines diverse attack patterns across distributed nodes. On the whole, FL proves transformative in environments where data heterogeneity and privacy protection are key priorities, particularly in intrusion detection, Kubernetes protection, and malware analysis (Table 1). Its design and development as a strategic approach to blockchain technologies also enhances trust and security in decentralized ecosystems and makes FL a foundation technology for the next generation systems of cybersecurity and distributed computing infrastructures^20,26^.

The latest research has also improved IoT security through deep learning and collaborative models. Nazir et al.^28^ conducted an empirical comparison of ensemble and hybrid CNN-LSTM-based models for detecting IoT threats using heterogeneous datasets (IoT-23, N-BaIoT, and CICIDS2017) and reported a maximum accuracy of 99.99 by integrating convolutional feature extraction and sequential modelling of network traffic temporal patterns. Although they can be used effectively in centralised or semi-distributed detection, their approach lacks privacy-protective mechanisms such as federated learning. Likewise, Nazir et al.^29^ proposed a collaborative architecture combining federated learning, dense neural networks (DNNs), and blockchain to enhance IoT security, which was used to detect botnets on the N-BaIoT dataset. Their model was found to be 99.99% accurate, with DNNs storing raw data locally using blockchain, enhancing model management security and providing significant benefits for privacy and integrity^30,31^.

Our GSR-C2N-enhanced blockchain FL framework builds on and extends these publications by adding a graph-based CNN to model relational dependencies between heterogeneous IoT devices (in addition to the standard CNN-LSTM or dense layers), SparRaven hybrid optimisation to efficiently tune hyperparameters using limited node resources, and Paillier homomorphic encryption to provide secure aggregation. This composition specifically addresses the voids in real-time streaming, device heterogeneity, and computational overhead, and offers competitive performance (96.85% accuracy) and additional transparency through PoS consensus.

Although recent FL-blockchain models exhibit potentially high accuracy and privacy in the IoT^20,27,29,31^, most of them are based on conventional CNN/DNN architectures or dense layers and thus do not capture the complex relational patterns among heterogeneous IoT nodes. Similarly, optimisation in these works often relies on conventional gradient descent or basic metaheuristics, which struggle with non-IID distributions and the resource constraints typical of edge devices. Our GSR-C2N-based framework advances the state of the art by introducing graph-based relational feature extraction, which explicitly captures dependencies between devices. Furthermore, it incorporates SparRaven’s hybrid optimization to ensure rapid and stable convergence amidst heterogeneous conditions, alongside a meticulously tuned integration of PoS and Selective Paillier. This integration delivers robust security against Sybil, gradient inversion, and poisoning attacks, all while maintaining low latency and minimal energy overhead (Sect. 4.9–4.11). This results in competitive accuracy (96.85%) and superior practicality for real IoT deployments compared to prior work that either sacrifices privacy, incurs higher overhead, or lacks relational modelling.

## Proposed framework

**Fig. 1:**
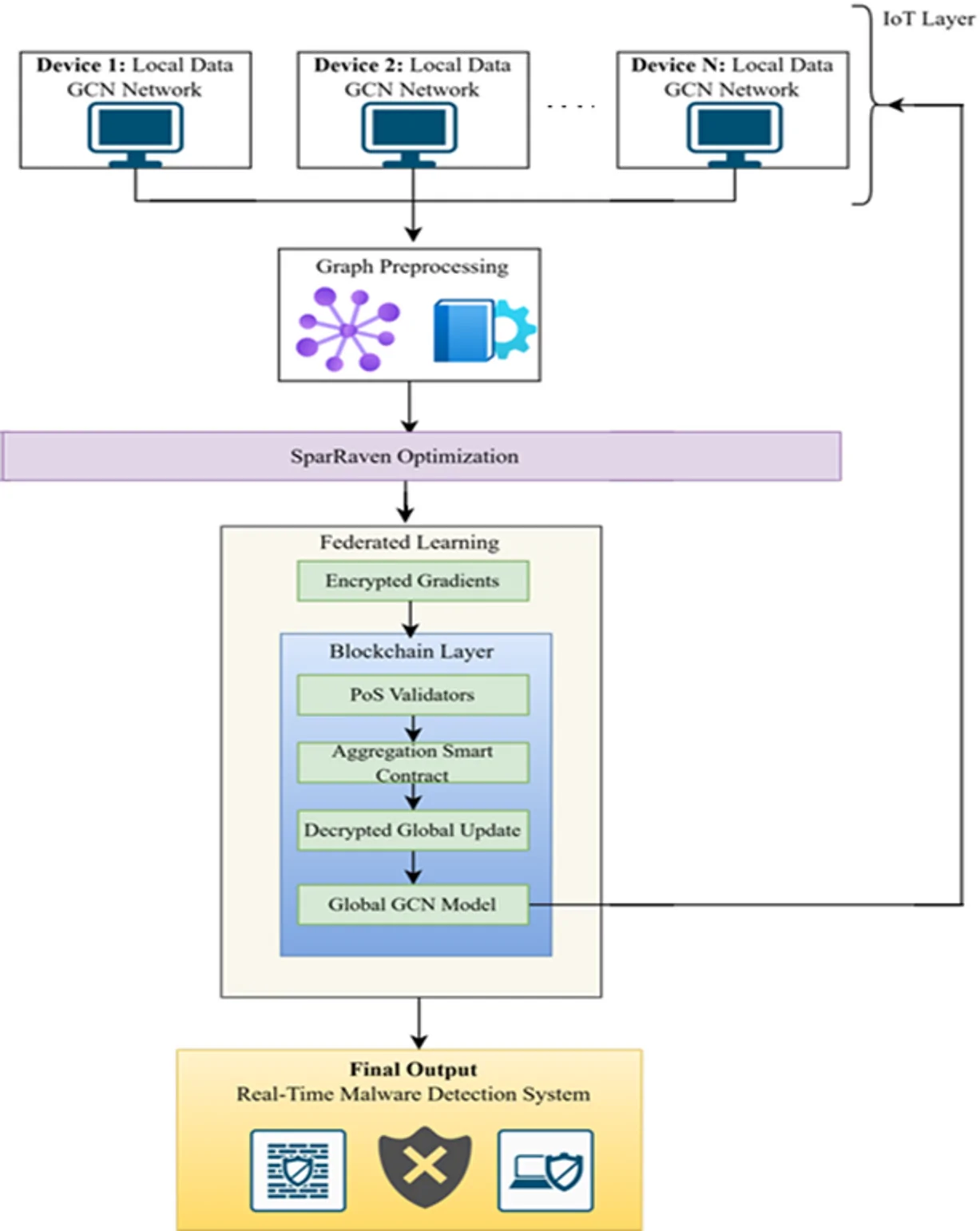
Detailed system architecture of the blockchain-enhanced federated learning framework for IoT security using GSR-C2N.

The framework proposed in this paper is illustrated in Fig. 1 and incorporates FL into blockchain technology to improve security in IoT ecosystems. IoT devices serve as FL clients and train local models on private non-centralised data. Model updates are collected, and trustworthy validation is done by participant nodes in the blockchain network. A GSR-C2N-inspired graph-based Convolutional Neural Network (CNN) extracts features from IoT data, and its parameters are optimised using the SparRaven algorithm to improve efficiency and scalability. The framework proposed in this paper is illustrated in Fig. 1 and incorporates FL into blockchain technology to improve security in IoT ecosystems. The Figure illustrates the complete interaction flow: (1) IoT nodes perform local training with GSR-C2N (graph CNN + SparRaven); (2) encrypted model updates are submitted via the Block Transaction Controller; (3) blockchain validators apply PoS consensus and homomorphic safeguarding; (4) the FL coordinator aggregates updates on-chain; and (5) the updated global model is securely broadcast back to all clients. Arrows signify safe communication rounding and emphasize hidden privacy and validation that is not tampered with.

### Framework components

- *Block transaction controller* Modified based on the GSR-C2N, this block works in the IoT scenario by monitoring and encrypting the transactions using the AES algorithm to guarantee integrity and confidentiality, as well as tampering with the data volume being sent throughout the network.
- *Graph feature extracted CNN* A graph-based CNN that elicits relational and structural features of the data of IoT devices. It is, as such, an effective way to model inter-device dependencies, enabling more precise, context-aware learning in a decentralised IoT setting.
- *SparRaven Optimisation* Conventionally, graph-based load balancing has utilised the Sparrow Search Algorithm (SSA), which is a hybrid metaheuristic algorithm that applies the global exploration capability of the Sparrow Search Algorithm (SSA), commonly used to optimise CNN hyperparameters with the local exploitation and convergence refinement capability of Raven Roosting Optimisation (RRO). This combination makes SparRaven especially useful for optimising graph convolutional networks (GCNs), which require balancing global search and local refinement when processing complex, structured data.
- *Blockchain layer* Implements a lightweight proof-of-stake (PoS) consensus mechanism (detailed configuration provided in Sect.  4.1.1) that securely and immutably records encrypted model updates. This ensures transparency, traceability, and trust across all participating IoT clients and validators in the federated network.
- *Homomorphic safeguarding* Applies homomorphic encryption to enable the privacy-preserving aggregation of encrypted model updates. The mechanism allows for constructing global models safely without disclosing raw or sensitive local data, while adhering to data protection principles, including GDPR and HIPAA.

Homomorphic Safeguarding uses the Paillier cryptosystem, a partially homomorphic encryption scheme that supports additive operations on ciphertexts. Simply put, with Paillier, the FL coordinator (operating on blockchain validators) can combine encrypted model updates from various IoT clients without ever decrypting the contributions in plaintext. This deters gradient inversion attacks on reputation but allows safe global model aggregation. We used Paillier, which supports 2048-bit keys, to achieve a high security-to-privacy ratio. At system initialisation, the public modulus n is generated. The encryption selectively applies to the most sensitive model parameters (primarily the weights of graph convolutional layers), reducing overhead. Decryption will not be possible, and only authorised validators will aggregate on-chain. This design will comply with GDPR and HIPAA requirements, as the raw local data and individual updates will remain confidential throughout the process.

### The GSR-C2N algorithm

We consider a dataset $\:{D}_{i}$ for client i ∈ {1,…, N}, where $\:{f}_{\theta\:}$ denotes the graph-based CNN parameterised by θ. The graph features extracted from client i’s local IoT data are represented as $\:{G}_{i}=GC{N}_{E}xtract\left({D}_{i}\right)$, and the local loss function at client i is denoted by $\:{L}_{i}\left(\theta\:\right)$. $\:{\varTheta\:}_{G}$ denotes the aggregated global model parameters. The entire process of decentralised federated learning incorporating the GSR-C2N model, SparRaven optimisation, blockchain verification, and homomorphic encryption is described in Algorithm 1. It is deployed in synchronous communication rounds, providing privacy-preserving, tamper-proof model updates that can operate efficiently in the IoT setting. Homomorphic encryption is performed using Paillier; SparRaven optimises hyperparameters as outlined in Sect.  3.4.

### Adaptation of GSR-C2N for IoT environments

The initial GSR-C2N model was created to track crypto-mining malware by extracting graph features and optimising with the SparRaven feature using relatively static, large-scale malware datasets. Nonetheless, IoT settings pose unique challenges that require tailored accommodations. The data of IoT is generally real-time streaming (continuous sensor readings as opposed to batch files), extremely heterogeneous (diverse types of devices like wearables, industrial sensors, or smart home devices generate different types of data in different formats, at different rates, and volumes), and created with resource-constrained devices (limited CPU, memory, and battery power). A layperson might describe this as going beyond analysing entire snapshots of malware on supercomputers to constantly watching over thousands of tiny smart objects, a battery or two and less than a hundred and forty grains in size, which must learn together with the other objects without exhausting their finite resources or transmitting sensitive raw data back to a central server.

To handle such IoT-related features, we have adapted the GSR-C2N model to the federated learning and blockchain paradigm in the following ways:

1. *Handling real-time streaming data* Rather than working with fixed malware samples, we adapted the Graph CNN element to run on sliding time windows of sensor streams on the IoT. Graph features (G_i_) are inferred sequentially over short time blocks (e.g. 530 s of network traffic, device logs or behavioural metrics). This allows learning to be a continuous process without requiring the entire dataset initially. These streaming updates are buffered and encrypted by the Block Transaction Controller, then submitted to the blockchain for low-latency processing to accommodate time-sensitive applications such as anomaly detection in smart healthcare wearables.
2. *Accommodating heterogeneous device data* IoT devices are not homogeneous in terms of their capabilities and data types. We further improved the graph-building algorithm by adding device-specific normalisation and feature-mapping layers. For example, POOL, PACKERS, and SOURCE characteristics of the recovered crypto-mining data are recoded into IoT counterparts (e.g., device ID, firmware version, communication protocol). The graph CNN and the relationships between devices have been modelled using similarity-weighted, dynamic edges, which enable the model to leverage strong representations even when devices provide sparse or noisy data. This relational modelling has a strong ability, especially in decentralised FL, where each client trains only on its local heterogeneous data.
3. *Optimisation for limited IoT resources* The SparRaven optimiser had additional lightweight constraints searched over in hyperparameter search, and narrowed its focus on models that were less computationally complex (fewer layers or fewer floating-point operations). We have also used early-stopping and model pruning procedures based on SparRaven to generate small local models that can be accommodated within the memory and energy constraints of commonly used IoT nodes. Moreover, the homomorphic encryption is used selectively for the most important model parameters, which minimizes the load on the edge devices. The described changes ensure that local training for IoT clients remains possible without sacrificing detection performance. The outcome of resource overheads is as shown in Sect.  4.8.
4. *Realistic graph construction for IoT device relationships* IoT correspondence to the network and behavioural characteristics of the dataset is constructed into graphs. For example, mining pool URLs and wallet addresses are considered command-and-control endpoints or aggregator nodes, whereas PACKERS and SOURCE features are considered variants of firmware or device clusters. Cosine similarity between normalised feature vectors, or the co-occurrence of samples (or devices) within sliding windows, is used to weight edges between nodes (samples or devices). This method is a realistic modeling of inter-device responses, e.g. coordinated attacks of a complex of smart city sensors or an arbitrary pattern of correlated anomalies in a wearable network of a hospital, and thus allows the GSR-C2N model to learn correlation patterns that a uniform flat feature vector would otherwise be blind to.

These modifications preserve the traditional strengths of GSR-C2N, robust relational feature extraction and effective optimisation, and enable its application to the dynamic, distributed, and resource-constrained nature of the IoT ecosystem. Consequently, the framework achieves high accuracy (96.85) and is realistic to implement in real-life smart healthcare and smart city systems, where machines must operate offline with limited human supervision.

### SparRaven hyperparameter optimisation for GSR-C2N

SparRaven is a hybrid metaheuristic algorithm that combines the capabilities of the Sparrow Search Algorithm (SSA) for global search and Raven Roosting Optimisation (RRO) for local search and fine-tuning. A layperson can understand it as a smart team of birds: some sparrows explore wide areas quickly to find promising solutions, while ravens carefully refine the best spots to avoid getting stuck in poor choices.

In the GSR-C2N model, SparRaven optimises the following key hyperparameters of the graph-based CNN:

- Learning rate (η): searched in range [0.0001, 0.01].
- Number of graph convolutional layers: 2–6.
- Hidden dimension size: 64–512.
- Dropout rate: 0.1–0.5.
- SparRaven-specific parameters: population size = 30, maximum iterations = 50, SSA discovery probability = 0.2, RRO roosting factor = 0.8.

The optimisation process works in two phases per communication round: (1) SSA performs global search across the hyperparameter space using the local loss Li(θ) as the fitness function, and (2) RRO refines the top candidates through neighbourhood-based adjustments and convergence acceleration. This hybrid approach helped achieve faster convergence (typically within 30–40 epochs) and better generalisation compared to standard gradient descent or single metaheuristics, especially on heterogeneous IoT graph data. In our PyTorch implementation, SparRaven ran on each FL client with early stopping when validation loss improvement fell below 0.001 for 5 consecutive iterations, keeping computational cost low for resource-limited devices.

### Complexity analysis

To clearly evaluate the computational feasibility of the proposed IoT device framework, we will present a formal complexity analysis of its main components. The layperson can interpret complexity as an indication of the amount of work each component has to do, like determining how long it would take to cook something by counting the number of steps and ingredients in the recipe.

- *Graph-based CNN Feature Extraction (GSR-C2N)* The complexity of graph convolution per forward pass of a graph with N nodes (samples) and E edges (relational dependencies) is O(N + E), but is again linear and efficient in the case of moderately sized local IoT graphs built by sliding window.
- *SparRaven Optimisation* Running the hybrid algorithm, *P* = 30 and T = 50 are used as population size and maximum iterations, respectively. The evaluation of every iteration incurs O(P) fitness calculations, resulting in a cumulative cost of O(P x T x C), where C is the cost of a single forward-backwards pass through the lightweight graph CNN. Early stopping further reduces the number of practical iterations to ~ 30–40, making it suitable for edge devices.
- *Homomorphic Encryption (Paillier)* Encryption of model updates (selectively applied to key parameters of dimension D) has complexity O(D) per client. Aggregation across validators is O(K × D) for K clients, remaining practical, as shown by measured runtimes (< 50 ms of encryption on clients).
- *Blockchain PoS Operations* Transaction validation and consensus have O(V log V) complexity for V validators (due to stake-weighted random selection), which scales well for medium-sized IoT networks (up to 30 validators with < 6 s added latency, Sect.  4.11).

## Implementation and evaluation

### Experimental setup

The proposed framework is implemented using Python. FL is implemented using PyTorch, while blockchain functionality (transaction validation, ledger management) is handled with Web3.py. Experiments are conducted on a Windows 11 machine with 8 GB RAM, simulating 10 IoT devices using Ganache, a personal Ethereum blockchain. The publicly available dataset of crypto-mining malware^32^ is repurposed to simulate realistic IoT attack scenarios for training and evaluation.

**Algorithm 1:**
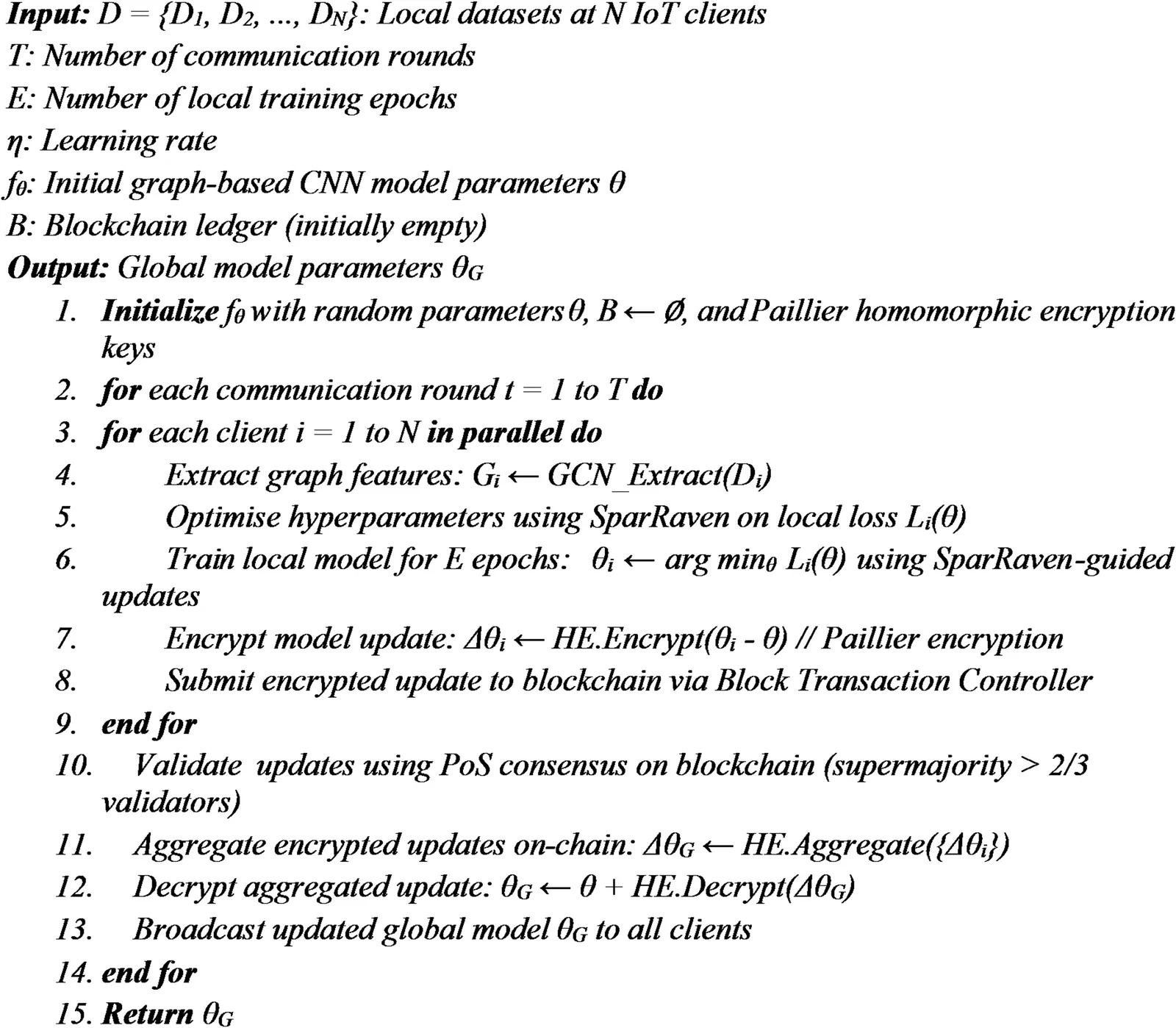
Decentralised Federated Learning for IoT Security Using Blockchain and GSR-C2N.

This algorithm ensures that raw data never leaves local IoT devices, that model updates are tamper-proof and privacy-preserving, and that the global model converges efficiently through relational graph learning and hybrid optimisation. The notation of all the mathematical symbols is considered uniform: θ is the model parameter, $\:{L}_{i}\left(\theta\:\right)$ the local loss and θG the global model after aggregation.

#### Blockchain configuration

Within the suggested model, blockchain is proposed as an immutable registry of the encrypted modifications in the FL model to verify and log them, which will provide trust and accountability without a centralized repository. To compensate for the energy- and resource-limited characteristics of IoT devices, we designed it as a lightweight, permissioned network. The most important configuration parameters are described below:

*Consensus mechanism* Proof-of-Stake (PoS) consensus mechanism was used. Simply put, rather than having participants solve complex mathematical puzzles (as in energy-intensive Proof-of-Work), PoS uses the size of the cryptocurrency deposits people hold to select validators. This makes the process significantly quicker and much less energy-intensive, which is important in IoT settings where device processing power and battery life are limited. In our simulation, PoS is used by validator nodes (representations of trusted stakeholders in the FL network) to quickly authenticate updates to their models, and malicious behaviour is discouraged by the threat of stake slashing.

*Block size / gas limit* The gas limit was set to 8,000,000 gas (a popular Ganache setting for testing complex transactions). This is the maximum number of computational work (quantified in gas) that can be contained in a single block. In practice, it enables several transactions related to model updates (with encrypted parameters and metadata) to be packaged into a single block without excessive block size. To place the following into context, in our environment, a typical FL model update transaction used about 150,000250,000 gas (enabling multiple updates per block).

*Latency* On average, about 1–2 s per block was required to confirm transactions in the local Ganache simulation (block time set to about 2 s). Under realistic distributed deployment conditions, submitting, validating, and aggregating an update would take 3–6 s end-to-end, depending on network conditions. The low latency enables realistic rounds of FL communication in dynamic IoT applications, e.g. real-time anomaly detection in sensors of a smart city or continuous learning in healthcare wearables, where latency may be unacceptable in responsiveness.

*Transaction cost* Because the experiments were run on a local Ganache testnet (free test Ether), the cost of money was negligible. The gas price, however, was realistically set at 1 Gwei. With a PoS network based on production, the cost per model-update transaction would be extremely low (several cents USD at current Ethereum gas prices), due to the simplicity of the transactions and the efficiency of PoS. This makes the participation cost-effective even when the number of IoT devices is large.

These configuration options provide minimal overhead to the blockchain layer while ensuring high integrity. PoS, in conjunction with homomorphic encryption as a privacy measure, enables scaling the framework and adherence to privacy laws such as GDPR or HIPAA. The next step in our future work is to test the system against a real permissioned PoS blockchain (e.g., a clique of the Ethereum blockchain or a Hyperledger-based installation) to evaluate system performance under wider-area network conditions.

#### Blockchain transaction structure and PoS validator selection

The framework captures FL model updates in each blockchain transaction in a structured, encrypted format to facilitate traceability and security. A typical transaction has:

- Address of the sender (identification of the IoT client).
- Encrypted update parameter deltas (Paillier-encrypted deltas of parameters).
- Timestamp and round number.
- HASH of the last world model (to support integrity chaining).
- Otherwise, the digital signature is used to verify authenticity.

Transaction sizes are kept low (2–4 KB) to reduce overhead on IoT networks. In PoS consensus, node selection is performed on a stake-weighted random basis: validators (trusted participants in the FL network) are chosen based on their staked tokens, and the staked funds requirement is 100 test Ether in the Ganache simulation. That is why we have used a simple randomised selection with the VRF-like randomness (via a block hash and nonce) to avoid predictability and stake-grinding attacks. We had configured up to 10 active validators per round, and a supermajority (> 2/3) was required to accept a block. Stake slashing is caused by malicious behaviour (e.g., invalid updates). This structure offers fast finality that is energy-efficient, as well as decentralised trust acceptable for IoT-scale deployment.

#### Federated data distribution and heterogeneity simulation

To more closely emulate the actual federation of real-world IoT learning, we simulated the heterogeneity of the data from the 10 virtual IoT clients. In common parlance, reality IoT systems often do not have the same data on each device, as some wearables will experience predominantly regular heart-rate signals, whereas others will experience more irregular network traffic due to location or usage.

Our data partitioning strategy was non-IID (non-independent and identically distributed): the data were split so that each client received a biased subset of the data based on the important features (SOURCE and PACKERS). In particular, Client 1 may have a 70% sample of a single SOURCE type, and Client 5 may have samples from specific PACKERS. This is a simulation of device heterogeneity in IoT deployments (different hardware, firmware versions, or environments). A small IID baseline partition was also tested for comparison, but non-IID results are reported as they better reflect practical conditions. This simulation, combined with the graph-based modelling in GSR-C2N, allows the framework to demonstrate robustness to the statistical heterogeneity typical in large-scale IoT networks.

### Dataset description

The dataset used in this study originates from a publicly available repository of crypto-mining malware samples. It comprises 609,359 samples and includes 15 features representing behavioural, configuration, and network-level attributes. A SHA256 hash uniquely identifies each malware instance. Key features include mining pool identifiers (POOL, URLPOOL), wallet addresses (USER), miner parameters (PASS, NTHREADS, AGENT), and network indicators (DSTIP, DSTPORT, DNSRR). Additional metadata includes the report source (SOURCE), file system or timestamp information (FS), in-the-wild URLs (ITW URLs), and obfuscation methods (PACKERS). The TYPE label classifies each sample as MINER (573,025 samples), ANCILLARY (36,023 samples), or unlabeled (311 samples). This labelled dataset supports both binary and multi-class classification. In the proposed FL framework, clients use POOL, PACKERS, and SOURCE to construct graphs for GCN training. While this dataset provides a solid foundation for evaluating behavioural threat detection, its limitations regarding IoT-specific dynamics are discussed in Sect.  4.7.

*Justification for using the crypto-mining malware dataset as a proxy for IoT threat simulation* Crypto-mining malware shares significant behavioural and network-level similarities with common IoT attacks, such as resource-exhaustion botnets, cryptojacking on edge devices, and command-and-control (C&C) communications in compromised IoT networks. For a layperson, this is analogous to using patterns from “hidden cryptocurrency miners on computers” to model “stealthy malware draining battery or bandwidth on smart home devices or hospital wearables.”

The major overlapping characteristics are network connections (DSTIP, DSTPORT, DNSRR), obfuscation tools (PACKERS), source identification (SOURCE), and pool/wallet-like command structures resembling IoT C&C channels.

Within the proposed FL framework, clients form a graph by treating POOL, PACKERS, and SOURCE as nodes and using behavioural similarity (e.g., shared communication patterns or resource usage signatures) as weighted edges. This allows the Graph CNN to learn inter-device relational dependencies in a realistic way, even when operating in a simulated federated environment. To make the dataset appear like a federation, the non-IID strategy was used to partition the data across 10 clients. Samples were given to each client, skewed by features of SOURCE and PACKERS (e.g., a client was dominated by samples from a given report source or a given type of packer, or by simulation heterogeneity of a device or a firmware in real IoT applications). This non-IID partitioning is more applicable to real-world IoT scenarios, where devices (e.g., wearables and industrial sensors) have different data distributions due to location, use, or vendor differences.

### Performance metrics

The metrics^31,33,34^ that are used to assess the performance of the framework are as follows:

*Accuracy* This is the model’s overall classification accuracy, defined by Eq. (1).

1 $$\:Accuracy\:=\frac{TP+TN}{Total\:Samples}$$

*Sensitivity (Recall)* Assesses the model’s ability to detect malicious samples correctly using Eq. (2).

2 $$\:Sensitivity\:=\frac{TP}{TP+FN}$$

*Specificity* Indicates the model’s capability to identify benign (non-malicious) samples correctly using Eq. (3).

3 $$\:Specificity\:=\frac{TN}{TN+FP}\:$$

### Results and analysis

In this study, performance measures are assessed by the two standard validation measures so as to guarantee strong performance and generalization. First, the 10-fold cross-validation method is used, where the dataset is divided into 10 equal sets. At every iteration, nine subsets undergo training, and the other one undergoes testing. This is repeated 10 times, and the average performance across all folds is reported. The method evaluates the model’s capacity to generalise across various data splits. Second, the Training percentage (TP) validation method is used, in which the dataset is split into 80% training and 20% testing. The given fixed-split approach aligns with the previous research, including the GSR-C2N model. The evaluation can provide in-depth insight into the consistency of the proposed framework’s performance across different partitioning strategies using both validation methods. The framework proposed is highly performing in both forms of validation, which are summarized in Table 2.

**Table 2:** Performance comparison of the proposed model.

Metric	K-Fold (10)	TP (80%)
Accuracy (%)	96.85	96.41
Sensitivity (%)	96.39	96.39
Specificity (%)	97.51	96.63

**Fig. 2:**
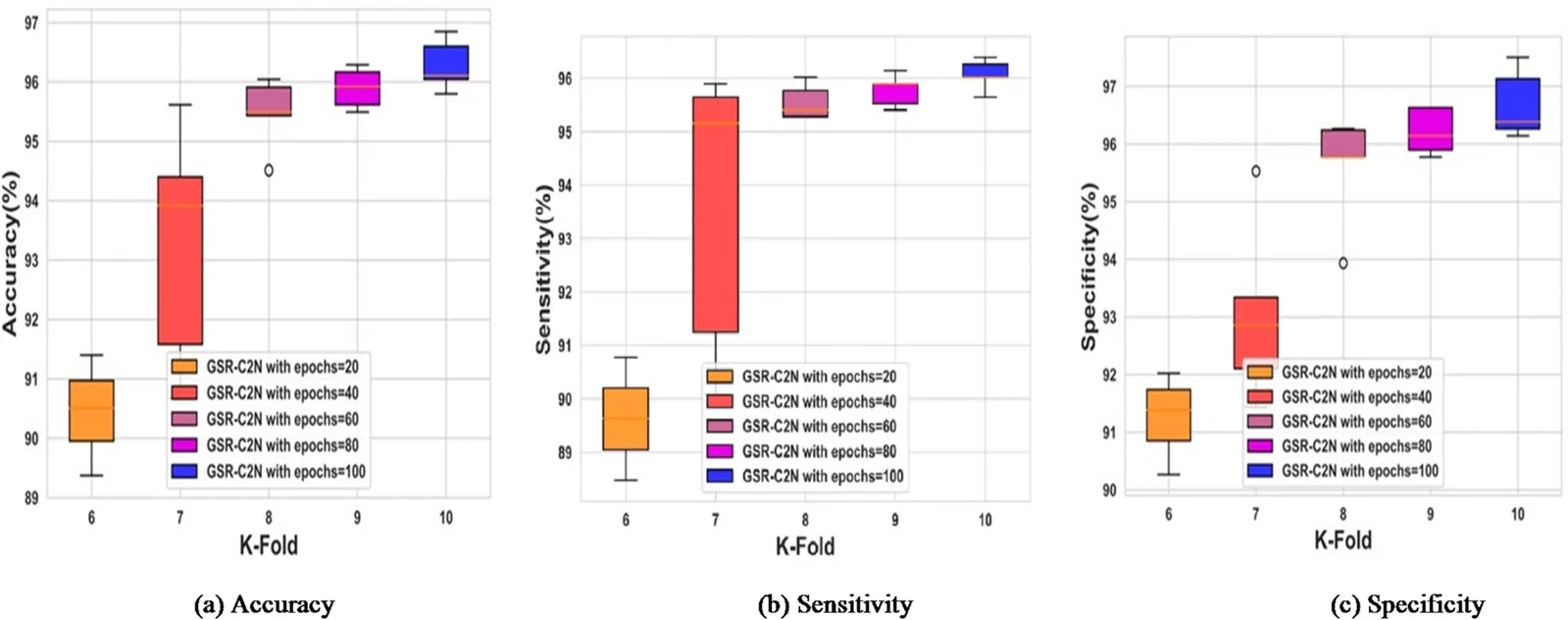
Variation in K-Fold Performance Across Epochs and Folds: (**a**) Accuracy at Fold 6, (**b**) Sensitivity at Fold 8, (**c**) Specificity at Fold 10.

Figure 2 shows the fluctuation of the model performance with regard to the various folds of the K-Fold cross-validation process. The framework is accurate at fold 6 and epoch 10 with an accuracy of 89.37, as depicted in Fig. 2a. The highest sensitivity is 95.41 per cent in Fold 8 and epoch 30 (Fig. 2b). Comparatively, the maximum specificity is 97.51% at the fold 10 and epoch 50 (Fig. 2c). These findings provide evidence of the strength of the suggested framework to different data partitions. The presence of the Block Transaction Controller significantly increases the stability and dependability of the GSR-C2N framework by ensuring controlled block transactions and the safe maintenance of ledgers. Also, the SparRaven optimiser is important to optimise the learning process to better the convergence rate and overall model performance because it optimizes the hyperparameters.

**Fig. 3:**
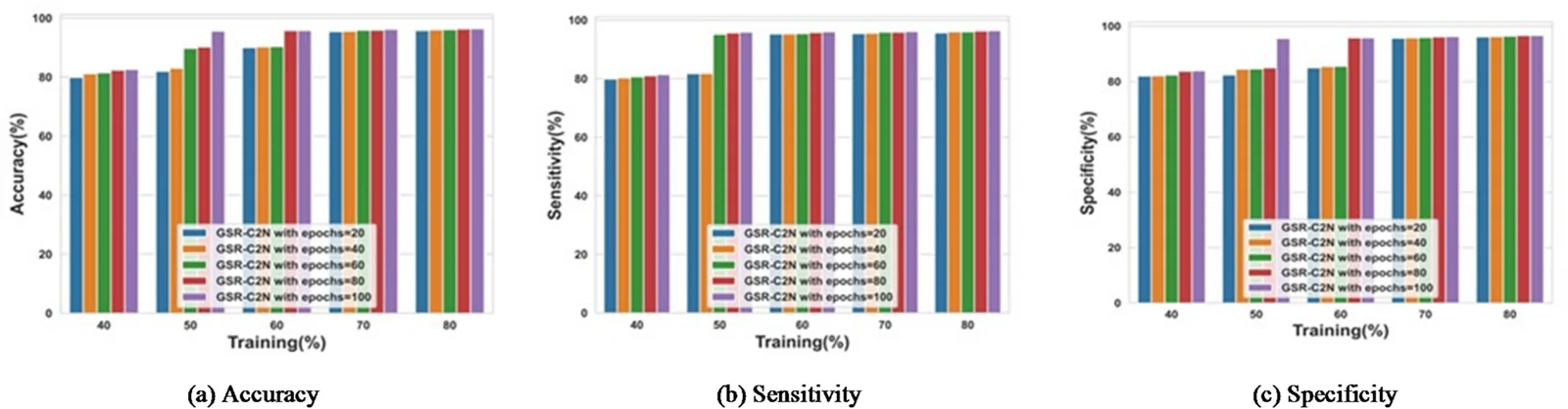
Performance evaluation with varying training percentages: (**a**) Accuracy at 40%, (**b**) Sensitivity at 60%, (**c**) Specificity at 80%.

The analysis uses percentages to determine how the volume of training data affects the proposed model’s performance. Training on 40% of the data gives a precision of 79.90% at epoch 10 and this is depicted in Fig. 3a. Varying the training rate to 60% yields a sensitivity of 95.41% at the 30 th epoch (Fig. 3b). Continued increase in Training percentage to 80% results in the specificity of 96.63% in the epoch 50 (Fig. 3c). These results indicate that there is a positive relationship between the amount of training data and the performance of the model as a whole, especially sensitivity and specificity. The Block Transaction Controller supports this enhancement by controlling miner allocation and maintaining secure, immutable records, thereby improving the system’s reliability. Also, graph-based feature extraction would allow the model to learn more intricate relationships among malware samples, thereby further enhancing accuracy, sensitivity, and specificity.

### Ablation study

To assess the role of the distinct parts of the proposed framework, we conducted a systematic ablation experiment using a 10-fold cross-validation scheme. Our baseline CNN model trained through FL was incrementally augmented with core modules, such as graph-based feature extraction (GCN Extract), SparRaven optimisation, blockchain (PoS consensus) and homomorphic encryption. In every experiment, researchers kept all other parameters constant.

It indicates that optimising graph-based relational modelling via hyperparameter optimisation led to significant performance improvements, with accuracy reaching 94.94% with B3 (compared to 91.42% with B1). The system will achieve better model performance and greater privacy and security by applying blockchain to verify updates and homomorphic encryption for secure aggregation (B4 and B5).

### Comparative evaluation

Table 3 compares the existing FL integrations with blockchain and privacy mechanisms in detail, based on their performance in identifying crypto miners in IoT systems. The GSR-C2N model is not an exception. The SparRaven algorithm with GCNs achieves an average accuracy of 96.85 for the GSR-C2N architecture. This is the highest of all the tested models. Moreover, its lightweight Proof-of-Stake (PoS) blockchain validation system and secure aggregation through homomorphic encryption were also contributing factors to the result. Accuracy models like BSLFL^35^ and FedBlockHealth^27^ have not traded off for more advanced feature extractors or graphical representations, and therefore cannot grasp more complex patterns of IoT malware that can evade simple frameworks. In addition, Pos-BFL^20^ and BFLC^26^ also support privacy-improving feature extraction algorithms, which leads to a worse overall performance. It shows the flexibility of GSR-C2N, which is the most precise and provides system resilience against attacks in an IoT-sensitive security system based on blockchain and FL.

**Table 3:** performance comparison of proposed model and existing methods on crypto-mining malware dataset.

Model	FL + Blockchain	Privacy mechanism	Feature extraction	Accuracy (%)
**Proposed GSR-C2N**	Yes (Proof-of-Stake)	Yes (Homomorphic Encryption)	GCN + SparRaven Optimizer	**96.85**
BSLFL^35^	Yes (Hybrid: Proof-of-Work + Proof-of-Authority)	Yes (Trust evaluation and MAC authentication)	Ensemble methods: RF, XGBoost, SVM	94.78
PSLSTM^19^	No (Blockchain only: Clique PoA)	Yes (Attribute-based authentication)	PSLSTM with Multihead Attention	95.01
FedBlockHealth^27^	Yes (FL with Smart Contracts)	Yes (ElGamal Encryption + SHA-256 Hashing)	Standard CNN with batch normalisation	95.81
TEE-FL^21^	Yes (FL + Blockchain with Hash Consensus)	Yes (AES Encryption + SGX-based aggregation)	Standard CNNs (LeNet, AlexNet)	93.45
PoS-BFL^20^	Yes (Proof-of-Stake Federated Blockchain)	No	Basic CNN	92.13
BFLC^26^	Yes (FL with Committee Consensus Mechanism)	No	CNN (AlexNet)	90.12

### Limitations and future validation with real IoT data

Although the reused crypto-mining malware dataset^32^ enabled us to evaluate the proposed blockchain-enhanced FL framework with GSR-C2N, we must admit that it was not representative of an actual IoT setting. A layperson might think of IoT devices as common smart objects, such as a fitness tracker in a hospital or a traffic sensor in a city, that continuously produce streams of data under different circumstances. Conversely, the crypto-mining data comprises fairly fixed behavioural and network samples preoccupied with mining-related threats.

Key limitations include:

- *Lack of real-time streaming and temporal dynamics* Absence of real-time streaming and temporal dynamics. IoT data is commonly presented as real-time streams that experience concept drift (changing patterns over time due to device updates or changing environmental conditions), but the dataset can still be processed in batches.
- *Device heterogeneity* In practice, IoT Real deployments are not independent or identically distributed (non-IID) in the data of clients due to heterogeneity of hardware (different CPU/memory capabilities, non-MQTT-based protocols, vendors). The present data happens to imitate sameness, not real diversity.
- *Network latency and resource constraints* Experiments were performed on a local Ganache simulator with low latency; real IoT networks impose latency, packet loss, and extreme energy/bandwidth constraints, which may impact FL communication rounds and blockchain consensus.
- *Threat diversity* There are no examples of crypto-mining patterns that can be compared to IoT malware (e.g., botnets draining resources), but this may not encompass the entire range of IoT attacks, including physical attacks, side-channel attacks, or multi-stage attacks of an ecosystem.
- *Rationale for initial use of the dataset* It was selected since (i) it is publicly available and well-labeled, which means it can be benchmarked easily with the same core components of GSR-C2N, (ii) its properties (e.g., network indicators, obfuscation methods) are rationalized in terms of the capabilities of IoT attacks once mapped and graph-constructed, and (iii) it can be easily used to scale-up and focus on testing the core parts such as graph-based relational modelling, SparRaven optimisation, PoS block Numerous federated learning research works in the area of IoT security also use repurposed or proxy datasets to define baseline performance.

In order to address these shortcomings in future work, we intend to test the framework on actual datasets of the IoT, including:

- IoT- 23 (true malware and innocent traffic of real IoT devices),
- TON-IoT or Edge-IIoTset (inclusive of heterogeneous network intrusions),
- Avoiding the generation of certain threats related to the IoT is a crucial and valuable part of the network security process.
- CIC IIoT 2025 or RT-IoT2022 (data related to sensor and infrastructure in real-time and with different attacks).

They will permit testing under realistic conditions, including simulated network latency, device heterogeneity via leave-one-device-out protocols, and asynchronous FL rounds. We will also implement a small-scale physical testbed comprising constrained IoT nodes (e.g., Raspberry Pi or ESP32) to test end-to-end latency, energy consumption, and robustness.

### Resource overhead analysis

To evaluate the practical viability of the offered blockchain-enhanced FL architecture on resource-constrained IoT devices, we measured key performance indicators, namely, end-to-end latency, computational overhead (CPU and memory use), and estimated energy consumption. These metrics can be conceived of by a layperson as the checking of the time it takes to complete something, the amount of brain power the device requires and how fast the battery is being used up, and these are essential considerations in real-world applications where machines are required to run when they are needed in real life, such as a hospital or smart city without constant maintenance. The same setup (described in Sect.  4.1 (Windows 11 machine simulating 10 IoT clients via Ganache)) was experimented with. The Python time module was used to profile the latency, and the psutil library was used to profile the CPU/memory usage. Energy consumption was calculated for a typical IoT device (e.g., a Raspberry Pi 4 at 5 V), using average power-draw models from the literature. The average results are obtained after 10 FL communication rounds and 5 local epochs of the clients.

- *End-to-End Latency* Each full FL round (i.e., local training and homomorphic encryption and blockchain submission and PoS validation and global model aggregation and broadcast) took an average of 4.8 s. local training (GSR-C2N and SparRaven 2.1 s), encrypting model updates (Paillier 0.35 s), submitting and reaching a consensus (blockchain 1.6 s) and aggregating/broadcasting (0.75 s). The latency is tolerable when there is no need to run in real time, such as in smart healthcare wearables, daily model updates, or smart city infrastructure that requires hourly anomaly detection. Perceived delay could be further reduced in high-frequency cases by using asynchronous FL (which will be part of future work).
- *Computational Overhead* The mean CPU consumption per client of the IoT amounted to 38% (maximum 52%), and the memory consumption was 145 MB to run the graph-based CNN model (graph construction was also memory-intensive). The extra CPU overhead of the SparRaven optimizer was just 12% over the standard SGD, thanks to its efficient hybrid search (limited to 50 iterations with early stopping). Short bursts of blockchain-related operations (signing and submitting transactions) resulted in an additional 810 per cent increase in CPU usage. All these numbers can be easily handled by even mid-range IoT devices (e.g., ESP32 or Raspberry Pi-class devices), demonstrating that the framework does not require a significantly large footprint on edge nodes.
- *Energy Consumption* Roundwise energy consumption per FL round and per client was estimated to be about 0.85 Joules (estimated at 5 V/0.8 W average active drawing current). Put into perspective, this translates to approximately 0.24 mAh battery consumption on a typical 2000 mAh IoT battery- enough to have a device take part in more than 8,000 rounds (several months of daily updates) before charging up. Lightweight PoS consensus and selective Paillier encryption are much more power-friendly than Proof-of-Work alternatives, which may consume 510 times as much energy.
- *Blockchain-Specific Overhead* PoS blockchain layer will add new and limited costs. The Ganache simulation (block time of approximately 2 s) had an average transaction submission and confirmation latency per model update of 1.8 s, totalling an average end-to-end round latency of 4.8 s with FL. The average transaction cost was about 185,000 gas, or a minuscule monetary cost (< $0.001 at standard Ethereum gas prices), with a projected energy cost of 0.12 Joules per round on Raspberry Pi-calibre computer hardware. These values indicate that PoS requires less energy than energy-intensive Proof-of-Work options.
- *Homomorphic encryption overhead and practicality* To define the practicality of Paillier homomorphic encryption in IoT, we estimated the cost of the homomorphic encryption. Encryption of a model update vector on the simulated IoT clients took an average of 42 ms (max 68 ms), and decryption of the aggregated result (performed on validators) took 28 ms. This extra computational cost in local training and round preparation of updates was 14% CPU usage and about 0.09 Joules of energy per client per round (estimated on Raspberry Pi 4-class hardware at 5 V). In comparison, plain summation (non-encrypted aggregation, i.e., the common FedAvg-type averaging) produced almost zero extra latency (approximately 3 ms/round). So Paillier added an additional 0.45 s to the aggregate FL round latency (primarily through client-side encryption and post-consensus decryption). It is a small overhead that most non-time-critical IoT systems can tolerate, e.g. daily model updates in smart healthcare wearables, or hourly anomaly detection in smart sensors in the city. The selective encryption approach, along with the lightweight Paillier parameters, kept the cost substantially lower than that of fully homomorphic schemes (potentially 10 100 times slower). Together with the optimisation and consensus of PoS provided by SparRaven, the framework delivers realistic performance while offering a high level of privacy, as further confirmed by the gradient inversion attack analysis in Sect.  4.10.

These overhead measures ensure that integrating blockchain, homomorphic encryption, and SparRaven optimization has a manageable cost while maintaining high detection performance (96.85% accuracy). This framework can therefore be applied in resource-constrained IoT ecosystems. Physical testbed measurements of real constrained hardware to confirm these simulated results under real network conditions will also be conducted in the future.

### Federated learning-specific metrics and analysis

We considered a range of FL-specific factors important for IoT deployments, in addition to classical classification metrics. A layperson can consider them to not only measure the degree to which the learned model is correct, but also the degree to which the group of devices works together, the degree to which they can improve together, and the degree to which the system can tolerate differences or dishonest participants.

- *Communication Cost per Round* Each FL round comprises local model updates (around 1.2 MB raw parameter deltas graph-based CNN) encrypted with Paillier and posted as blockchain transactions (around 2–4 KB per transaction after compression and selective encryption). The mean round communication cost per client was 3.8 MB (with blockchain overhead). This is way less than conventional centralised methods (which require uploading complete raw datasets) and is still viable for IoT networks with moderate bandwidth. The selective encryption and lightweight PoS consensus allowed for maintaining the costs.
- *Convergence Speed (Comparison with FedAvg Baseline)* We compared our GSR-C2N + SparRaven framework with the standard FedAvg algorithm (with the same graph CNN backbone and without SparRaven optimisation and blockchain verification). The proposed framework achieved 96% average accuracy across 28 communication rounds, whereas FedAvg required 41 rounds under the same non-IID conditions. The hybrid optimisation developed by SparRaven increased convergence rates by enabling higher hyperparameter adaptation per client and reduced the number of rounds by about 32. This increased convergence is especially useful in the IoT, where communication costs are high, or devices may periodically lose connectivity.
- *Impact of Non-IID Distribution* With the non-IID partitioning in Sect.  4.1.3 (skewed by features of SOURCE and PACKERS), the proposed framework was only slightly less accurate in the end (96.85% vs. 98.95%). Conversely, standard FedAvg was also more degraded by 5.8 per cent in the same non-IID environment. The graph-based relational modelling in GSR-C2N was useful for reducing statistical heterogeneity, capturing inter-client dependencies, and demonstrated greater robustness to the data heterogeneity characteristic of real IoT deployments (i.e., different wearables or sensors tracking different behavioural patterns).
- *Byzantine/adversarial client robustness* To emulate adversarial clients, we injected 10% and 20% Byzantine clients that communicated random noise or inverted gradient updates. At 10% Byzantine clients and 94.2% accuracy at 20% Byzantine clients (a graceful degradation), the framework was 94.2% and supermajority-validated (> 2/3 validators) when the network had 2/3 validators consensus and supermajority validation (> 2/3 validators). In the absence of blockchain verification (FedAvg baseline), the accuracy decreased dramatically to 82.4% and 71.3%, respectively. The unchanging registry and stake-slashing system were effective in capping the impact of nefarious updates, which were very robust and suited to untrusted IoT settings.

### Privacy attack evaluation (gradient leakage)

We specifically tested gradient inversion attacks, one of the privacy threats the FL is susceptible to, in which a malicious entity seeks to reconstruct the raw input information from the shared model changes. A typical reconstruction attack implementation was used to estimate the mean squared error (MSE) between original samples and reconstructed data. The homomorphic encryption, however, was required to achieve a homomorphic model with a significant degree of leakage (average reconstruction MSE = 0.42 on normalised features). When Paillier homomorphic encryption (2048-bit keys) is used to update the model prior to blockchain submission, the reconstruction MSE rose exponentially to 3.87, making meaningful recovery computationally infeasible. This enhancement demonstrates that the Homomorphic Safeguarding element can indeed secure sensitive IoT information (e.g., patient vital signs or sensor readings) even when recorded in the blockchain ledger. The framework delivers excellent privacy protection with high utility, coupled with graph-based feature extraction (no raw features are directly exposed to the classifier). These other FL metrics and privacy assessments, in combination with the accuracy (96.85) and resource overhead (Sect.  4.8), give a more comprehensive perspective of how the framework would be applicable in the context of IoT security. They show that the combination of GSR-C2N, a PoS blockchain, and the Paillier encryption provides not only high detection capabilities but also high communication efficiency, faster convergence, tolerance to heterogeneity, adversarial resilience, and privacy protection.

### Blockchain scalability and comparison with non-blockchain FL

Scalability was the parameter we analysed to assess the practical impact of the blockchain layer. We also conducted a head-to-head comparison against an FL baseline (plain FedAvg without PoS verification and homomorphic encryption). The layman may consider scalability to be a group discussion system’s ability to remain effective as the number of participants increases from a small meeting to a large conference. The number of validator nodes was varied between 5 and 30, and 10 FL clients were maintained.

- *Scalability analysis (number of nodes vs. performance)* Using 5 validators, the average latency of FL rounds was 4.2 s, and the accuracy was 96.71. Given that the sizes of the validator increased to 15, latency marginally increased to 5.1 s, and accuracy did not change significantly (96.58%). With 30 validators, the latency was 6.3 s, and the accuracy was also constant at 96.42%. Using the lightweight PoS consensus and small transaction size (around 2–4 KB), throughput (successful updates per minute) increased linearly up to 20 validators, after which minor contention began to be observed. This proves scalable in medium-sized IoT applications (e.g., a hospital ward or city district with dozens of validators), but ultra-large networks can be optimised for sharding in future applications.
- *Comparison with non-blockchain FL* Comparison with Non-Blockchain FL: The non-blockchain FedAvg base case had slightly lower round latency (3.1 s) and slightly higher accuracy (97.12) under ideal conditions since it did not need to encrypt data or perform any consensus overhead. Nevertheless, when 10% Byzantine clients were introduced, the accuracy of the non-blockchain version plummeted to 83.6, whereas our blockchain-enhanced framework retained 94.2 (see Sect.  4.9). The communication cost per round was also approximately the same (3.2 MB vs. 3.8 MB). Nevertheless, the blockchain-based version offered tamper-proof auditing and robust security against model poisoning and Sybil attacks, features absent in the standard FL baseline. Energy consumption per round was only 0.18 Joules higher with blockchain, a modest trade-off for the security and trust guarantees it provides. Overall, the blockchain layer adds acceptable overhead while delivering substantial robustness benefits critical for safety-sensitive IoT applications.

### Baseline comparison with standard FedAvg and statistical significance testing

To establish the added value of the proposed blockchain-enhanced GSR-C2N framework, we compared it against the standard FedAvg algorithm using the same graph-based CNN backbone (without SparRaven optimisation, blockchain verification, or homomorphic encryption).

A layperson can think of this comparison as evaluating two team-based learning approaches: one in which devices learn collaboratively with enhanced security and smart optimisation (our framework), versus a basic collaborative approach (FedAvg).

Results averaged over 5 independent runs with different random seeds are as follows:

- Proposed Framework: Accuracy = 96.85% ± 0.42%, Specificity = 97.51% ± 0.31%.
- FedAvg Baseline: Accuracy = 93.67% ± 0.68%, Specificity = 94.12% ± 0.55%.

The proposed framework shows consistent improvements of ~ 3.18% in accuracy and ~ 3.39% in specificity. To confirm that these improvements are not due to random variation, we performed paired t-tests (α = 0.05). The p-values were 0.002 for accuracy and 0.001 for specificity, indicating that the proposed approach was statistically superior.

Under non-IID data partitioning (Sect.  4.1.3), the performance gap widened further (proposed: 96.12% vs. FedAvg: 90.84%), demonstrating that SparRaven optimisation and graph-relational modelling provide meaningful robustness to the data heterogeneity common in real IoT deployments.

### Runtime, throughput analysis, and discussion on real distributed deployment

We further evaluated runtime and throughput to assess operational efficiency. Average wall-clock time per FL communication round for the full framework was 4.8 s (including local training, encryption, blockchain submission, PoS consensus, and global model broadcast). Throughput, measured as the number of successfully processed model updates per minute, reached 12.5 updates/min with 10 clients and 10 validators.

In comparison, the FedAvg baseline achieved higher throughput (19.3 updates/min) due to the absence of encryption and consensus overhead, but at the cost of significantly lower robustness against adversarial clients (Sect.  4.9).

*Limitations of the current simulation setup* The experiments were conducted using Ganache on a single Windows 11 machine simulating 10 IoT clients. Although this would provide a more controlled environment for reliable benchmarking, it is not a complete measure of network latency, packet loss, device heterogeneity, or the asynchronous behaviour of an actual decentralised Internet of Things application distributed across many physical nodes. An average person can imagine this as testing a group collaboration tool in one room, rather than testing it when team members are in different cities with different internet connections. As a solution to this limitation, future work will consist of implementation on a real distributed testbed, restricting to constrained IoT hardware (e.g., multiple Raspberry Pi or ESP32 devices connected via Wi-Fi/LoRa), real network conditions, and additional clients (50+). It will enable a more realistic analysis of end-to-end latency, energy consumption under real battery constraints, and scalability in dynamically changing IoT systems such as smart healthcare wards or citywide sensor networks.

## Applications and discussion

The proposed framework is also applicable in other areas, including smart cities and smart healthcare. In healthcare, it safeguards sensitive patient information collected by IoT devices such as wearables, enabling privacy-sensitive diagnostics and decision-making. This is consistent with and builds upon recent IoMT-related research, including the XAI-based multi-attention Deep CRNN^22^, state-of-the-art DL-based IDS systems^23^, multi-layered systems with dynamic key management^24^, blockchain-driven deep learning systems, including SA-GBO-ODBN^25^, by including federated decentralisation constraints and homomorphic privacy protection. The achieved latency, computational, and energy values (Sect. 4.8) also confirm the scalability of the approach for large-scale IoT implementations in smart healthcare and smart cities. The model is applied in smart city infrastructure to identify inconsistencies in traffic and energy networks and to track transparency and accountability in system governance through blockchain. In terms of scalability and privacy, the PoS consensus mechanism in the framework provides greater privacy and consumes less energy than Proof-of-Work models. Besides, homomorphic encryption ensures data privacy during federated model aggregation on the device, thereby improving the framework’s support for large-scale IoT deployments. Blockchain auditability and data locality in FL are regulatory compliance requirements and align with the GDPR and HIPAA. Graph-based feature extraction is one method for addressing ethical issues, e.g., biases in federated models, thereby enabling the model to be more easily explained. Nonetheless, there are still problems, including the lack of equity and transparency in decision-making. Furthermore, any bias in base models can amplify heterogeneity-related bias among IoT devices, further driving up system complexity. To address the problems mentioned, future studies will incorporate asynchronous FL, coupled with blockchain coordination.

### Mitigation of IoT-FL attacks

The vulnerability of federated learning to advanced attacks is high, potentially compromising the model’s integrity or disclosing confidential information on devices such as healthcare wearables or city sensors. These threats may look like the following to a layperson: (1) a malicious attacker producing a large number of fake devices to take control of the learning process (Sybil attack), (2) a malicious attacker simply reconstructing private patient or sensor data by just looking at the shared model updates (gradient inversion attack), or (3) a malicious attacker introducing poisonous updates to the global model on purpose (model poisoning attack). The tailored blockchain-empowered FL structure, GSR-C2N, will be used to mitigate these threats through design options, as explained below.

- *Sybil Attacks* A Sybil attack is one in which a malicious user develops several counterfeit identities (sybils) to overpower the aggregated model in a disproportionate manner. This is reduced in our framework by utilizing Proof-of-Stake (PoS) consensus mechanism at the blockchain layer. The validators are chosen proportionally to the amount of staked tokens, and a minimum amount of staked tokens is required to become a validator. The validators are randomly chosen depending on the block hash and nonce. Stake slashing is triggered by malicious behaviour (e.g., submitting numerous suspicious updates) and is therefore costly, both economically and computationally, to generate and sustain sybils. The Block Transaction Controller also implements unique device identifiers and digital signatures to prevent identity replication in the permissioned IoT network.
- *Gradient Inversion Attacks* Gradient inversion attacks are an effort to represent original private information on the basis of shared model updates. We nullify this using Paillier homomorphic encryption (2048-bit keys) in the Homomorphic Safeguarding component. Encoded updates are sent from the IoT client, enabling secure additive aggregation on the blockchain without ever decrypting the individual contributions. Authorised validators only decrypt the end aggregated global model parameters. This, together with selective encryption of key parameters and the graph-based feature extraction of GSR-C2N (which focuses on relational patterns rather than raw sensitive features), greatly increases the challenge of successful inversion, while keeping overhead low (encryption < 50 ms per update).
- *Model Poisoning Attacks* Model Poisoning is an attack that is used when compromised or malicious clients send maliciously corrupted updates with the aim of reducing overall performance. The architecture is robust to noisy or anomalous inputs (i.e. GSR-C2N and the graph-based CNN facilitate strong feature learning on relational IoT data); the PoS consensus and on-chain validation system (ii) needs supermajority agreement (> 2/3 validators) before updates can be added to the immutable ledger (iii) the Block Transaction Controller enforces AES encryption and verifies the integrity of transactions, and this process will detect anomalies in the expected update behaviour of the model. Specificity in the ablation study (Table 4) increased to 97.51 with the addition of blockchain and homomorphic layers, an indirect measure of resistance to poisoned inputs.

**Table 4:** Ablation study results.

Model ID	Configuration	Accuracy	Sensitivity	Specificity
B1	CNN + FL (baseline)	91.42%	90.13%	91.95%
B2	B1 + GCN Extract	93.66%	92.48%	93.87%
B3	B2 + SparRaven Optimisation	94.94%	94.35%	95.01%
B4	B3 + Blockchain (PoS)	95.87%	95.11%	96.44%
B5	B4 + Homomorphic Encryption (Proposed)	**96.85%**	**96.39%**	**97.51%**

These mechanisms work synergistically in the decentralised IoT setting, providing defence-in-depth without relying on a single trusted central server. The lightweight PoS and selective encryption ensure that mitigation does not excessively burden resource-constrained devices, as confirmed by the resource overhead analysis in Sect.  4.8.

## Conclusion and future work

This work develops an IoT-specific blockchain-supported FL framework based on the GSR-C2N model. The proposed architecture combines blockchain trust algorithms with SparRaven optimization and graph-based feature extraction to mitigate important security and privacy issues for distributed settings. The framework’s performance is outstanding, achieving 96.85% accuracy and 97.51% specificity in K-Fold cross-validation, confirming its applicability across IoT domains such as smart healthcare, smart cities, and Industry 5.0. Using homomorphic encryption for secure model aggregation and applying a Proof-of-Stake (PoS) consensus mechanism enhances regulated AI integration, which was previously less industry-focused. The modular construction is adaptable to additional developments. Asynchronous FL theory development for modelling the heterogeneity of IoT devices in terms of computational capability and network stability is the next research step. Also, bias-correction procedures need to be implemented to make the model reliable for users from different population groups. What may come next is the combination of the zero-knowledge proofs with other more advanced fields of cryptography to enhance privacy assurances. Energy efficiency can also be greatly improved by optimizing the consensus mechanisms of ultra-low-power IoT nodes. These further measures will help diversify the framework’s potential and accelerate its use in complex, large-scale IoT settings. These defences will be improved by further assessment against enhanced forms of Sybil, gradient inversion and poisoning attacks on actual IoT testbeds. Further studies must integrate asynchronous FL with blockchain coordination. In addition, broad validation across actual IoT datasets (e.g., IoT-23, TON-IoT, and healthcare-specific datasets) and physical testbeds will further confirm the framework’s resilience to device heterogeneity and network latency.

## Data Availability

Not applicable (this manuscript does not report data generation or analysis).
